# Diet impact on mitochondrial bioenergetics and dynamics

**DOI:** 10.3389/fphys.2015.00109

**Published:** 2015-04-08

**Authors:** Rosalba Putti, Raffaella Sica, Vincenzo Migliaccio, Lillà Lionetti

**Affiliations:** Department of Biology, University of Naples “Federico II”Naples, Italy

**Keywords:** mitochondrial fusion, mitochondrial fission, dietary fat, caloric restriction, energy balance

## Abstract

Diet induced obesity is associated with impaired mitochondrial function and dynamic behavior. Mitochondria are highly dynamic organelles and the balance in fusion/fission is strictly associated with their bioenergetics. Fusion processes are associated with the optimization of mitochondrial function, whereas fission processes are associated with the removal of damaged mitochondria. In diet-induced obesity, impaired mitochondrial function and increased fission processes were found in liver and skeletal muscle. Diverse dietary fat sources differently affect mitochondrial dynamics and bioenergetics. In contrast to saturated fatty acids, omega 3 polyunsaturated fatty acids induce fusion processes and improve mitochondrial function. Moreover, the pro-longevity effect of caloric restriction has been correlated with changes in mitochondrial dynamics leading to decreased cell oxidative injury. Noteworthy, emerging findings revealed an important role for mitochondrial dynamics within neuronal populations involved in central regulation of body energy balance. In conclusion, mitochondrial dynamic processes with their strict interconnection with mitochondrial bioenergetics are involved in energy balance and diet impact on metabolic tissues.

## Mitochondrial dynamics and bioenergetics

Mitochondria are referred to as the “powerhouses” of the cell due to their prominent role in ATP production and cellular metabolism regulation. Together with their energetic role, mitochondria are very dynamic organelles that continuously divide, collide and fuse with other mitochondria. Therefore, their morphology is highly variable. It can shift between small round punctuated structures or reticulum networks of elongated mitochondria as a result of the balance between fusion and fission processes. Various work has been done to analyze the mechanism of this dynamic behavior and different proteins (a group of large ATPases) have been identified as being involved (James et al., 2003; Ishihara et al., 2004, 2006; Jagasia et al., 2005; Liesa et al., 2009). In particular, the main proteins involved in mammalian mitochondrial fusion processes are mitofusin 1 and 2 (Mfn1 and 2) and autosomal dominant optic atrophy-1 (Opa1). Mitofusin is located on the outer mitochondrial membrane and plays a role in the outer membranes fusion, whereas Opa1 is located on the inner mitochondrial membrane and is involved in the inner membrane fusion process (Malka et al., 2005). In addition, Opa1 controls cristae remodeling and protects from apoptosis (Frezza et al., 2006). It should be noted that also Mfn2 has a dual role, since other than its well-known role in mitochondrial fusion (Zorzano et al., 2010), Mfn2 is also implicated in the structural and functional connection between mitochondria and the endoplasmic reticulum (ER) and it may play a role in ER stress development in conditions of metabolic stress (de Brito and Scorrano, 2008, 2009). As regards fission processes, dynamin related protein 1 (Drp1) and fission protein 1 (Fis1) are the main proteins involved. Drp1 is located mainly in the cytosol and is recruited on the outer mitochondrial membrane by Fis1 that is inserted on the outer membrane (Yoon et al., 2003; Santel and Frank, 2008). Drp1 is also involved in the regulation of apoptosis (Frank et al., 2001).

A correct balance between fusion and fission processes is important for mitochondrial bioenergetics. It has been suggested that mitochondrial fusion processes are induced in conditions in which an optimization of mitochondrial bioenergetics is required, whereas fission processes are associated with mitochondria degradation and therefore they are induced under conditions in which mitochondria are damaged (Westermann, 2012). Deficiency in proteins involved in mitochondrial fusion (Mfn2 and Opa1) reduce respiration in several cell types (Liesa et al., 2009). Mfn2 repression is associated with decreased substrate oxidation and cellular metabolism (Pich et al., 2005). Alterations in OPA1 expression also affect mitochondrial bioenergetics, for example Opa1 depletion causes a reduction in basal respiration (Chen et al., 2005). Therefore, emerging evidence suggests that the balance between mitochondrial fusion and fission processes play an important role in the regulation of mitochondrial energetics.

Several recent reviews analyzed the cellular roles of mitochondrial dynamics and the molecular mechanisms of fusion and fission (McBride et al., 2006; Detmer and Chan, 2007; Hoppins et al., 2007; Liesa et al., 2009; Westermann, 2010, 2012; Elgass et al., 2013; Zhao et al., 2013; Lackner, 2014; da Silva et al., 2014), the present review focused on the impact of diet on mitochondrial dynamic behavior and function in the main metabolic tissues, such as liver and skeletal muscle, as well as the involvement of mitochondrial dynamic processes in body energy balance regulation.

## Mitochondrial dynamics and bioenergetics in obesity

It is well known that mitochondrial dysfunction plays an important role in obesity related diseases, such as insulin resistance and non-alcoholic fatty liver diseases. It has also been shown that impairments of mitochondrial function are associated with changes in mitochondrial network in the main tissues involved in obesity related diseases. Indeed, in obese and insulin resistant Zucker rats, skeletal muscle is characterized by a reduced glucose uptake, insulin resistance and reduced oxygen consumption accompanied by a reduction in Mfn2 expression, mitochondrial size and in the extent of the mitochondrial network (Bach et al., 2003). Reduced Mfn2 expression was also reported in skeletal muscle of obese type 2 diabetic patients. A decreased mitochondrial proton leak and increased bioenergetics efficiency in Mfn2-depleted cells has been demonstrated (Bach et al., 2003). Therefore, it has been suggested that Mfn2 loss-of-function found in obesity conditions enhance bioenergetics efficiency and contribute to obesity development by reducing energy expenditure and increasing fat energy store (Liesa et al., 2009). In line with this suggestion, conditions characterized by increased energy expenditure (such as cold exposure, administration of β3 adrenergic agonist, chronic exercise) are associated with higher Mfn2 expression in skeletal muscle and brown adipose tissue (Cartoni et al., 2005; Soriano et al., 2006). Furthermore, an increase in mitochondrial fission proteins was reported in skeletal muscle in mice with genetically induced obesity (ob/ob) as well as in mice with high fat diet-induced obesity (Jheng et al., 2012). Mitochondrial dysfunction was associated with enhanced fission processes in liver from db/db mouse, animal model of obesity and insulin resistance (Holmström et al., 2012). All these reports suggest that a shift toward fission processes is linked to mitochondrial dysfunction in the main tissues, such as skeletal muscle and liver involved in obesity related metabolic disease. In line with this suggestion, our group recently published data on the impact of high fat diet on mitochondrial function and dynamic proteins content in rat skeletal muscle and liver. In particular, we analyzed the effect of two different fat dietary sources (saturated vs. polyunsaturated omega 3) on the above parameters (Lionetti et al., 2014).

### Impact of dietary saturated fatty acids

**Figure 1:**
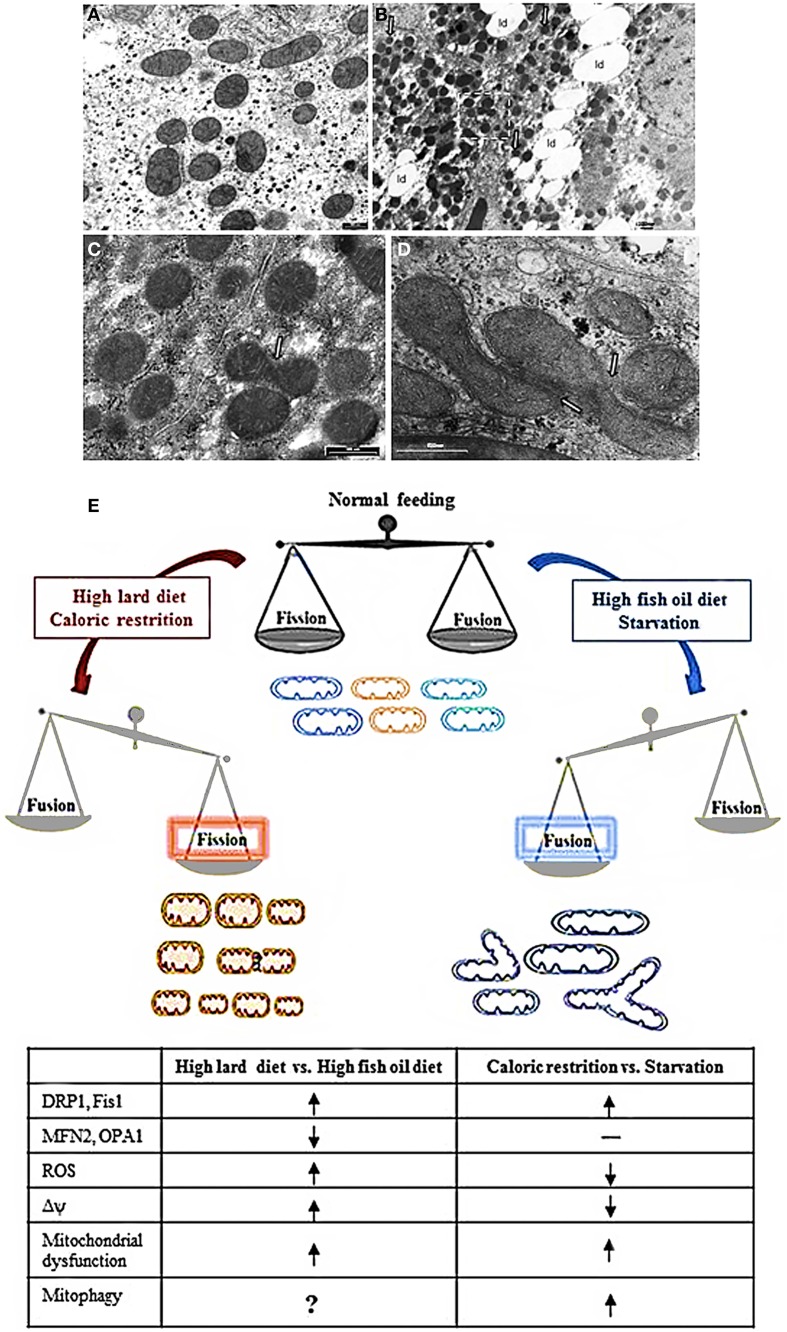
**Mitochondrial morphology and dynamics. (A–D)** Electron micrographs of ultrathin rat liver sections. **(A)** section of hepatocytes of rat fed a control diet. The mitochondria showed normal features with tubular, ellissoidal and round profiles. **(B)** section of hepatocyte of rat fed a HL diet. Note the abundant lipid droplet (ld) in cytosol and the prevalence of small mitochondria with round profile. **(C)** Higher magnification of insert in B showing an image suggesting a fission process (arrow). **(D)** section of hepatocyte of rat fed a HFO diet showing a cluster of mitochondria in which the fusion process is ongoing (arrows). **(E)** Diet impact on mitochondrial dynamics and function. See text for details. Shift toward fission processes has been found in response both to HLD and CR. However, in response to HLD, fission processes are associated to impairment of mitochondrial function and oxidative stress and may be useful to improve substrate intake in mitochondria or remove damaged mitochondria. On the other hand, in response to CR, mitochondrial fragmentation is associated to mitochondrial biogenesis useful to the maintenance of ATP level to meet cellular energetic needs. In addition, during CR, oxidative stress is reduced and mitochondrial fission may contribute to remove damaged mitochondria by mitophagy explaining the prolongevity effect of CR. Noteworthy, in condition of a more stressed nutrient deprivation, such as cell starvation, it has been reported an increase in mitochondrial fusion processes with elongated mitochondria that were spared by mitochondrial degradation and contributed to increase ATP production to face energetic needs. Fusion processes were found to be increased also in condition of HFO diet, where they contributed to an increase in mitochondrial substrate oxidation and to avoid lipid accumulation and oxidative stress. HLD, high fat diet rich in lard; HFO, high fat diet rich in fish oil; Δψ, mitochondrial membrane potential; ROS, reactive oxygen species; CR, caloric restriction 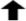
= increase, 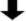
= decrease; —= no changes.

High fat diet rich in saturated fatty acids (high lard, HL, diet) elicited hepatic fat accumulation and insulin resistance, which was parallel to impaired mitochondrial function, increased reactive oxygen species (ROS) production and a dysregulated expression profile of mitochondrial dynamics proteins (Lionetti et al., 2014). In particular, HL diet induced an increase in mitochondrial fatty acid utilization, but a decrease in FADH_2_ linked oxygen consumption and in fatty acid induced proton leak, which caused an increase in mitochondrial energetic efficiency and an increase in ROS content. As regards to mitochondrial dynamics proteins, HL diet elicited a decrease in Mfn2 and an increase in protein involved in fission processes (Drp1 and Fis1) accompanied by the presence of numerous small round mitochondria vs. control diet, as observed by electron microscopy (Figures 1A–C,E) (Lionetti et al., 2014).

As regards to skeletal muscle, our results suggest that an HL diet also induced a shift toward fission as observed by electron microscopy and by immunureactivities analysis (Lionetti et al., 2013) which showed weak signal for fusion proteins and strong signals for fission proteins in rats which were fed an HL diet (Lionetti et al., 2013). These findings are in line with previous reports that Mfn2 expression is reduced in skeletal muscle of obese Zucker rats and type 2 diabetic patients (Bach et al., 2005; Hernández-Alvarez et al., 2010). In addition, it has also been suggested that Mfn2 plays a role as a regulator of *in vivo* insulin sensitivity and may be a potential target in diabetes drug development (Sebastián et al., 2012). Indeed, Mfn2 deficiency in mice produces mitochondrial dysfunction, increases oxidative stress and endoplasmic reticulum stress and activates JNK impairing insulin signaling in liver and skeletal muscle (Sebastián et al., 2012). This study suggested a role of Mfn2 in coordinating mitochondria and endoplasmic reticulum function, leading to modulation of insulin signaling *in vivo*.

Moreover, saturated fatty acids have also been reported to induce fission processes *in vitro* in differentiated C2C12 skeletal muscle cells (Jheng et al., 2012) associated with mitochondrial dysfunction. *In vivo*, as previously reported, smaller mitochondria and increased mitochondrial fission machinery have been described in the skeletal muscle of mice with genetic obesity and those with diet-induced obesity (Jheng et al., 2012). Moreover, inhibition of mitochondrial fission improved the muscle insulin signaling and systemic insulin sensitivity of obese mice (Civitarese and Ravussin, 2008).

Mitochondrial fragmentation and increased fission processes in association with ROS formation have also been reported after treatment with high glucose (HG) in both a rat liver cell-line and myoblasts (Yu et al., 2006). Such fragmentation has been suggested to provide metabolically active organelles with increased total surface area that would increase accessibility of metabolic substrate to carrier proteins (Yu et al., 2006). Therefore, it can also be suggested that HL diet induced-mitochondrial fragmentation might be an adaptive cellular response to increase mitochondrial intake and oxidation of surplus of dietary fatty acids, which would result in elevated ROS production (Lionetti et al., 2014).

### Impact of omega 3 polyunsaturated fatty acids

Different sources of dietary fats have been suggested to have different effects on mitochondrial function and dynamic behavior. In fact, in contrast to the effect of saturated fatty acids, omega 3 polyunsaturated fatty acids have been reported to improve mitochondrial function, reduce ROS production, and promote mitochondrial fusion both *in vitro* and *in vivo* experiments. In an *in vitro* steatotic hepatocyte model both eicosapentaenoic and docosahexaenoic acids increased the expression of Mfn2 and the ATP levels, and decreased oxidative stress (Zhang et al., 2011). On the other hand, in Mfn2-depleted steatotic hepatocytes, omega 3 PUFA did not induce the previously described effects (Zhang et al., 2011).

These data obtained *in vitro* were in line with our data showing improvement of mitochondrial function, reduced ROS production and induction of hepatic mitochondrial fusion through fish oil i*n vivo* (Lionetti et al., 2014). Indeed, the comparison of the effect of high fat diet rich in fish oil (HFO diet) and HL diet in rats showed that HFO diet led to less lipid accumulation in liver and higher fatty acid utilization. We also observed in HFO diet fed rats a mild mitochondrial uncoupling due to enhanced expression of uncoupling protein 2. These decreases in mitochondrial efficiency might contribute to increased fatty acid utilization and reduce ROS production. In HFO diet fed rats, a shift toward fusion was found with concomitant increases in Mfn2 and Opa1 as well as decreases in Drp1 and Fis1, in line with an increased number of tubular mitochondria observed by electron microscopy compared to HL diet (Figures 1D,E). This fusion phenotype was in accordance with reduced weight gain found in HFO diet vs. HL diet fed rats. With the limitation that the cause-consequence relationship between the leaner phenotype of HFO diet vs. HL diet fed rats and mitochondrial dynamics is not known, it can be suggested that the specific dietary fatty acid composition may play a role in obesity and hepatic steatosis development as well as in mitochondrial bioenergetics and dynamics (Lionetti et al., 2014).

Similar results were found in skeletal muscles of rats fed HFO diet, where, compared to HL diet, reduced fission processes and increased fusion events were suggested by immuoreactivity analysis and electron microscopy (Lionetti et al., 2013). Indeed, skeletal muscle sections from HFO fed rats showed a greater number of immunoreactive fibers for Mfn2 and Opa1 protein as well as weaker immunostaining for Drp1 and Fis1 compared to sections from HL fed rats. The shift toward fusion events in HFO fed rats was associated with the improvement of obesity and systemic and skeletal muscle insulin sensibility (Lionetti et al., 2013).

Differential effects of saturated and unsaturated fatty acids on mitochondrial morphology and dynamics were reported *in vitro* in C2C12 skeletal muscle cells. The results showed that treatment with saturated fatty acids induced mitochondrial fragmentations, whereas unsaturated and polyunsaturated fatty acids protected against palmitate-induced mitochondrial fission (Jheng et al., 2012).

## Starvation, caloric restriction and mitochondrial dynamics

Opposite mitochondrial dynamics behavior has been reported in two different conditions of nutrient deficiency, such as starvation (Rambold et al., 2011) and caloric restriction (CR) (Khraiwesh et al., 2013).

Mitochondrial elongation is a reversible response to nutrient deprivation in many cell culture types (Rambold et al., 2011). It depends on the type of nutrient depleted. Indeed, either glucose or serum elimination increased mitochondrial fragmentation, whereas mitochondrial fusion was induced by a nitrogen-source deficiency (either glutamine or amino acids). However, a combination of nutrient depletions induced a further mitochondrial elongation, suggesting that mitochondrial fusion can be modulated according to type and severity of starvation. Starvation-induced mitochondrial fusion depends on Mfn1 and Opa1 and is mediated by decreased DRP1 fission activity and by preventing Drp1 localization to mitochondria (Figure 1E). Mitochondrial fusion has a protective function, leading to an exchange of mitochondrial DNA and delaying apoptosis (Chen et al., 2010; Rambold et al., 2011). In fact, mitochondrial elongation might be useful to protect mitochondria from mitophagy. In accordance, during the initial period of starvation cytoplasmic components are degraded, whereas mitochondria become substrate much later (Kristensen et al., 2008), because mitochondria spared from degradation may contribute to maximize cellular energy production to sustain cell during nutrient deprivation (Rambold et al., 2011). Accordingly, mitochondrial fusion has been associated with increase in ATP production during stress and starvation (Gomes et al., 2011) producing beneficial effects for cells under conditions of low nutrient supply. Interestingly, it has been demonstrated that mitochondria provide membrane to autophagosomes during starvation (Hailey et al., 2010) and it can be also suggested that sparing mitochondria may be useful to permit them to serve as an autophagosome membrane source in nutrient depletion conditions.

On the other hand, in a study performed to evaluate dynamic mitochondrial behavior in an animal model of CR (mice submitted to 40% CR for 6 months), proteins related to mitochondrial fission (Fis1 and mitochondrial Drp1) increased, but no changes were detected in proteins involved in mitochondrial fusion (Mfn1/Mfn2, and Opa1) (Khraiwesh et al., 2013) (Figure 1E). A significant increase in the number of mitochondria per cells as well as in parameters related to mitochondrial biogenesis was also found in CR conditions (Nisoli et al., 2005; López-Lluch et al., 2006). Given that fission is the postulated mechanism for mitochondrial proliferation (Scheffler, 2007), the increase in Fis1 and Drp1 proteins support the idea of increased mitochondrial biogenesis in CR. Moreover, in a model of *in vitro* CR, the greater number of mitochondria was linked to reduced oxygen consumption and membrane potential (López-Lluch et al., 2006). As it has been demonstrated that ROS production by electron leakage increases at high membrane potential (Lambert and Merry, 2004), the decreased membrane potential found in CR conditions is in agreement with the lower ROS production associated with CR. Noteworthy, the levels of ATP production were no different in CR conditions vs. control (Khraiwesh et al., 2013). In effect, CR induced an increase in the number of mitochondria capable to maintain critical ATP levels in conditions of decreased oxidative stress. It is well known that CR attenuates age-dependent oxidative damage and it is correlated with an extension of life-span in animals as well as with prevention of cancer and diabetes (Sohal and Weindruch, 1996; Colman et al., 2009). It has been suggested that the increase in fission proteins found in CR may be useful in removing damaged mitochondria and to support the prolongevity effect of CR (López-Lluch et al., 2008; Khraiwesh et al., 2013). This suggestion seems to be in contrast with the report that unopposed mitochondrial fission in absence of mitochondrial fusion in the Δmgm1 mutants of S. cerevisiae (yeast Mgm1 is the ortholog of mammalian Opa1) leads to severe lifespan shortening (Scheckhuber et al., 2011). However, it should be noted that the different correlation found between mitochondrial fission processes and lifespan may be due to the different experimental model.

## Mitochondrial dynamics in regulation of energy balance

It is well known that in mammals the NPY/Agrp and POMC neurons within the arcuate nucleus of the hypothalamus regulate hunger and satiety. Recent works suggested that mitochondrial dynamics play an important role in these two neuronal populations, and that Mfn1 and Mfn2 are involved (reviewed in Nasrallah and Horvath, 2014). During positive energy balance (high fat diet exposure) in mice, mitochondrial fusion increased in orexigenic NPY/Agrp neurons to enable elevated neuronal activity and maximize storage of excess energy in fat (Dietrich et al., 2013). The electric activity of NPY/Agrp neurons was impaired when mitochondrial fusion mechanism was altered by cell-selectively knocking down MFN1 or Mfn2. The decreased activity of Agrp neurons was correlated with resistance to fat gain during high fat diet in Agrp-specific Mfn1 or Mfn2 knockout mice (Dietrich et al., 2013). Conversely in anorexigenic pro-opiomelanocortin POMC neurons, Mfn2 selective deletion causes severe obesity and leptin resistance (Schneeberger et al., 2013) probably by mediating ER stress-induced leptin resistance. In fact, ER stress plays a role in the development of obesity and leptin resistance (Zhang et al., 2008; Lionetti et al., 2009; Ozcan et al., 2009; Mollica et al., 2011) and genetic loss of Mfn2 generates ER stress (Sebastián et al., 2012). Indeed, Mfn2 plays an important role in the structural and functional communication between mitochondria and ER (de Brito and Scorrano, 2008). Schneeberger et al. (2013) showed that specific ablation of Mfn2 in POMC neurones causes a decrease in mitochondrial respiratory capacity and an increase in oxidative stress as well as loss of mitochondria-ER contacts, ER stress-induced leptin resistance, hyperphagia, reduced energy expenditure and obesity. Moreover, in diet induced obese mice, it was shown a decrease in mitochondrial network complexity and in mitochondria-ER association due to a reduction in Mfn2 expression in the hypothalamus which precedes the onset of obesity. On the other hand, Mfn2 overexpression ameliorates the diet induced obese phenotype (Schneeberger et al., 2013).

In all, these data suggested that mitochondrial dynamics, namely Mfn1 and Mfn2, in NPY/Agrp and POMC neurons may play a role in the central regulation of energy balance and in etiology of diet induced obesity.

## Concluding remarks

Mitochondrial function varies in accordance to cellular energetic needs and to nutrient supply. Noteworthy, a number of recent works have been focused on the importance of mitochondrial dynamic behavior in terms of fusion and fission processes in determining mitochondrial functionality in diverse diet conditions as well as in the central regulation of energy balance. Therefore, mitochondrial dynamic behavior contributes to bioenergetics physiological adaptation in response to the nutritional status (Figure 1E).

### Conflict of interest statement

The authors declare that the research was conducted in the absence of any commercial or financial relationships that could be construed as a potential conflict of interest.
